# Adhesive Capsulitis in Eight Dogs: Diagnosis and Management

**DOI:** 10.3389/fvets.2016.00055

**Published:** 2016-07-14

**Authors:** Brittany J. Carr, Sherman O. Canapp, Debra A. Canapp, Lauri-Jo Gamble, David L. Dycus

**Affiliations:** ^1^Veterinary Orthopedic and Sports Medicine, Annapolis Junction, MD, USA

**Keywords:** frozen shoulder, adhesive capsulitis, forelimb lameness, range of motion, fibrous scar tissue, synovitis

## Abstract

**Objective:**

To describe clinical and diagnostic findings as well as management of adhesive capsulitis in dogs.

**Background:**

Adhesive capsulitis, also known as frozen shoulder, is a syndrome defined by loss of range of motion of the shoulder and may be the end-stage manifestation of several primary conditions.

**Evidentiary Value:**

This is a case series report of eight dogs with chronic forelimb lameness diagnosed with adhesive capsulitis.

**Methods:**

Medical records (June 1, 2010–September 1, 2015) including, physical examination findings, radiographic findings, magnetic resonance imaging (MRI) findings, arthroscopy findings, and treatment plans were reviewed.

**Results:**

All dogs presented with a chronic, grade III–VI/VI forelimb lameness. On orthopedic examination, all dogs had moderate to significant discomfort on shoulder extension and flexion and severe restriction of range of motion. Six of the eight dogs had evidence of bone remodeling and sclerosis in the affected shoulder on radiographs. Six of the dogs had an initial diagnostic ultrasound performed, which revealed evidence of fibrous scar tissue. Five dogs had MRI performed that revealed moderate shoulder effusion and enhancement of the synovial lining of the shoulder. Arthroscopy was performed in five of the eight patients. Three were noted to have significant contracture, adhesions, and fibrous scar tissue of the joint capsule. Severe inflammation was noted throughout the synovium of two patients. All eight patients tried conservative management consisting of oral medications and rehabilitation therapy. Five of the eight patients received extracorporeal shockwave therapy. Three patients received regenerative medicine treatment in the affected supraspinatus and shoulder. Regardless of the treatment elected, none of the dogs were reported to have significant improvement.

**Conclusion:**

Adhesive capsulitis is an uncommon cause of chronic forelimb lameness. Further investigation is needed to describe the etiology and pathogenesis of adhesive capsulitis in dogs to evaluate the effectiveness of both non-surgical and surgical treatment modalities, establish treatment protocols, and evaluate short- and long-term clinical outcome of patients.

**Application:**

Adhesive capsulitis should be considered in patients with chronic forelimb lameness and moderate to significant discomfort and restriction on shoulder range of motion.

## Introduction

Adhesive capsulitis, also known as frozen shoulder, is a syndrome appreciated in human sports medicine and defined by loss of range of motion of the shoulder, impairing the patient’s ability to sleep, work, perform daily living activities, or desired recreational activities (1–5). Adhesive capsulitis may also be the end-stage manifestation of several primary conditions, including trauma, previous surgery, prolonged immobilization, endocrine disorders, or idiopathic causes (2, 6–12). Of patients affected by adhesive capsulitis, 70% are female and about 20–30% will develop the condition in the opposite shoulder (2). Only 20–30% of patients will report a history of minor trauma to the shoulder (6, 7), and it has been found to be more common in those with sedentary lifestyles (8). Regardless of the biological cause, adhesive capsulitis is defined as thickening and contracture of the joint capsule, which results in decreased intra-articular volume and capsular compliance so that glenohumeral motion is limited in all planes (2–4).

In humans, there are four stages associated with adhesive capsulitis, each based on the correlation of physical examination and arthroscopic examination of affected joint(s) and lasting about 6–9 months (1, 5). Stage 1 is characterized by pain with active and passive range of motion and a progressive loss of motion. Upon examination either under anesthesia or following intra-articular injection of local anesthetic, there is a significant improvement in range of motion to normal or minimal loss (13). Arthroscopic examination reveals a diffuse hypervascular glenohumeral synovitis, often most pronounced in the anterosuperior capsule (5). Histopathology reveals rare inflammatory cell infiltrates and findings consistent with a hypertrophic, hypervascular synovitis and a normal underlying capsule (14). Stage 2, also known as the “freezing stage,” is characterized by chronic pain and progressive loss of range of motion. Examination after intra-articular injection of local anesthetic or scalene block reveals relief of pain with only partial improvement in range of motion (13). Arthroscopic examination reveals a diffuse, pedunculated synovitis and a tight capsule with a dense feel on the insertion of the arthroscope (5). Histopathology reveals hypertrophic, hypervascular synovitis with perivascular and subsynovial scar formation and capsular fibroplasia (14). Stage 3, also known as the “frozen stage,” is characterized by minimal pain and significant shoulder stiffness. An intra-articular injection of local anesthetic or scalene block does not improvement in range of motion (13). Arthroscopic examination reveals a profound loss of capsular volume and remnants of fibrotic synovium that is not hypervascular (5). Histopathology reveals a dense, hypercellular collagenous tissue and a thin synovial layer without significant hypertrophy or hypervascularity (14). Stage 4, also known as the “thawing stage,” is characterized by minimal pain and progressive improvement in range of motion, resulting in capsular remodeling. Since these patients rarely undergo surgery, there is little arthroscopic or histological data available for patients with stage 4 adhesive capsulitis (13).

To the authors’ knowledge, there is only one paper reporting shoulder immobilization for 16 weeks in eight beagle dogs caused a loss of shoulder motion and focal capsular adhesion that resembled adhesive capsulitis seen in humans (15). Currently there is no other documentation of adhesive capsulitis in dogs in the literature. The purpose of this case series is to report clinical and diagnostic findings and management of adhesive capsulitis in dogs.

## Materials and Methods

Medical records (June 1, 2010–September 1, 2015) of eight dogs diagnosed with frozen shoulder were reviewed. Physical examination, diagnostic findings, arthroscopy findings, and treatment plans were reviewed. Inclusion criteria for patients were a diagnosis of frozen shoulder based on the presence of the following: history of unilateral thoracic limb lameness, severe restriction of shoulder extension (<110°) and flexion (>90°), and pain on manipulation of the shoulder. Patients were excluded from this study due to incomplete medical records or loss of follow-up.

### Diagnostic Imaging

Routine lateral and caudocranial radiographs of the shoulders were performed in all eight dogs and evaluated by a board certified surgeon. Magnetic resonance imaging (MRI) was offered to all patients and elected in five of the eight patients. An MRI of the affected shoulder was performed using a 1.5-T MRI unit. Musculoskeletal Diagnostic Ultrasound of the Shoulder was also offered to all patients and elected in six of the eight patients. If elected, patients were placed in lateral recumbency and the shoulder was clipped and prepared routinely. A 15.4 MHz linear probe was used to acquire longitudinal and cross-sectional images of the supraspinatus, biceps, infraspinatus, and teres minor tendons. The echogenicity, fiber pattern, and size of each tendon was recorded and compared to the contralateral, unaffected shoulder. The shoulder joint capsule was also imaged in the same cross-sectional image to assess the supraspinatus and biceps tendons. The echogenicity, integrity, and thickness of the joint capsule was recorded and compared to the contralateral, unaffected shoulder. The caudal humeral head was also assessed on ultrasound to confirm the presence of osteochondrosis or osteochondritis dissecans by placing the transducer in a longitudinal fashion parallel to the caudal humeral head.

### Shoulder Arthroscopy

A standard lateral portal was utilized for arthroscopic evaluation of the affected shoulder. Following aseptic preparation, a 22-gage needle was placed into the shoulder. Synovial fluid was aspirated using a 5-ml syringe to ensure the needle was in the joint. Approximately 5–10 ml of saline was then injected into the shoulder to distend the joint. A #15 blade was used to create a stab incision through the skin to allow for insertion of the cannula and trocar into the shoulder. The 1.9-mm arthroscope was then inserted into the cannula. Due to severe soft tissue contracture and joint space collapse, entry into the joint required fluoroscopic assistance in one patient. Another small 1-cm incision was made 3 cm caudal to the scope portal for insertion of instruments. Following arthroscopy, the incisions were closed routinely.

### Regenerative Medicine Therapy

Regenerative medicine therapy consisting of adipose-derived progenitor cell (ADPC) and autologous conditioned serum (ACS) in the affected shoulder and an ultrasound-guided ADPC and platelet-rich plasma (PRP) injection in any concurrently affected tendons was offered to three patients.^1^

Adipose tissue was collected from the falciform ligament within the abdomen during a routine ventral midline celiotomy. Approximately 20 g of falciform adipose tissue was collected and placed into a sterile container with Delbecco’s modified Eagle’s medium (DMEM) supplemented with fetal bovine serum (FBS), penicillin, and streptomycin. Approximately 30 ml of blood was collected from the jugular vein using an 18-gage butterfly needle into a syringe with 5 ml of CPDA anti-coagulant. Both the adipose and the blood samples were then prepared for shipment. Adipose and blood samples were shipped in validated containers at 4°C to Virginia Tech’s Regenerative Medicine Research Laboratory for processing. Adipose was processed using established techniques (33). The tissue was mechanically and enzymatically separated to release ADPCs. The cells were then washed in phosphate-buffered saline (PBS) and nucleated cells were counted and cultured in stem cell media at 37°C in a 5% CO_2_ incubator with 95% humidity. Adherent cells were monitored daily for adherence, growth, and phenotype. Once 70% confluent, cells were detached from the flasks, washed in PBS, and suspended in autologous PRP. PRP was prepared from anti-coagulated blood via centrifugation to obtain a fourfold increase in platelets and 80% reduction in white blood cells over whole blood. Platelet and white blood cell counts were performed for each sample to confirm appropriate concentrations prior to use. The samples were then shipped back to VOSM for injection at 4°C using validated shipping containers. Ultrasound guidance was used to administer ADPC–PRP therapy with a fenestration technique (15, 17).

## Results

### Population Sample

All patients included in this series were companion dogs. As described in Table 1, there were three mixed breed dogs, three Welsh Corgis, one German Shepherd Dog, and one Beagle. There were five male castrated dogs and three female spayed dogs. The mean age initial presentation was 8 years old (range 3–12 years old). All patients presented with a history of a chronic, unilateral forelimb lameness. One patient had a previous medical history of left medial patellar luxation, a left cranial cruciate ligament rupture and extracapsular suture repair, mitral valve insufficiency, and elevated liver enzymes. One patient had a previous medical history of hypothyroidism, which was controlled with medical management. The other six patients had no history of previous medical disease. Three patients had a history of previous trauma to the affected shoulder 12–18 months prior to presentation.

**Table 1:** **Sample population, history, and physical examination**.

Patient	Age (years)	Sex	Breed	Trauma	Lameness	Decreased ROM	Muscle atrophy	Other
1	3	MN	Welsh corgi	No	Chronic 4–5/6	Yes	Moderate	Increased abduction; pain left elbow
2	5	FS	Collie mix	No	Chronic 5–6/6	Yes	Severe	Swelling
3	10.5	FS	Maltipoo	Yes	Chronic 5–6/6	Yes	Mild	
4	11	FS	Beagle	No	Chronic 5–6/6	Yes	Severe	Mass cranial and medial to the shoulder
5	6	MN	German Shepherd	No	Chronic 3/6	Yes	Moderate	Increased abduction; “Loss of end feel” during the biceps stretch
6	7	MN	Mixed	No	Chronic 5/6	Yes	Severe	“Loss of end feel” during the biceps stretch
7	12	MN	Welsh corgi	Yes	Chronic 6/6	Yes	Severe	
8	8.5	MN	Welsh corgi	No	Chronic 5–6/6	Yes	Moderate	“Loss of end feel” during the biceps stretch

### Initial Orthopedic Examination

All eight patients had an initial orthopedic examination performed by a board certified surgeon (see Table 1). All patients initially presented with a chronic, grade III–VI/VI unilateral forelimb lameness (5 left forelimb, 3 right forelimb) (Table 2). On initial orthopedic examination, all dogs had moderate to significant discomfort on shoulder extension and flexion and restricted range of motion in the affected shoulder. In all patients, shoulder extension was <110° and shoulder flexion was >90°. Four patients were too painful to allow for range of motion or abduction of the affected shoulder. Pain and spasm on direct palpation of the supraspinatus and biceps tendon of the affected limb was noted in two dogs. Pain in the medial compartment of the elbow of the affected limb was appreciated in two dogs. There was moderate to severe muscle atrophy of the shoulder musculature of the affected limb in six dogs and mild muscle atrophy noted in two dogs. Soft tissue swelling and effusion of the affected shoulder was appreciated in one patient. In one patient, the biceps muscle felt contracted and shortened and significantly limited elbow extension was noted. In three patients, there was pain, spasm, and empty end feel during biceps stretch (pain with no mechanical or anatomic resistance in shoulder flexion with concurrent elbow extension). The remainder of orthopedic examination was within normal limits in all patients. At the time of initial consult, all patients were categorized to be in stage 2 of the 4 stages associated with adhesive capsulitis.

**Table 2:** **Lameness Numeric Rating Scale**.

Grade of lameness	Description
1	Lameness is not perceptible at a walk but perceptible at a trot
2	Lameness is perceptible at a walk and apparent at the trot
3	Lameness is apparent at both a walk and trot
4	Lameness is apparent at a walk and severe to non-weight bearing at a trot
5	Non-weight bearing lameness at a walk and trot
6	Unable to rise and walk

Different diagnostics were performed on each dog, including radiographs of the affected shoulder in all eight patients, diagnostic ultrasound in six of them, and arthroscopy in five of the eight patients. Five dogs had MRI performed of the cervical spine and affected shoulder. Arthrocentesis of the affected shoulder was performed in four of the eight dogs and submitted for cytology, whereas histopathology of the shoulder synovium was performed in only one patient. Results are described in Table 3.

**Table 3:** **Diagnostics performed**.

Patient	Radiographs	Ultrasound	MRI	Arthroscopy	Cytology and histopathology
1	Normal	Supraspinatus disruption; biceps tendon sheath effusion	Marked joint effusion and synovitis; damage to the supraspinatus tendon and medial glenohumeral ligament	Biceps tendinopathy; impingement of the biceps tendon; fraying and disruption of the subscapularis and of the MGL; disruption of joint capsule; grade III/IV cartilage erosion along the caudal humeral head and glenoid; RF treatment was performed	Mild mononuclear inflammation (76% large mononuclear cells and 24% small mononuclear cells)
					Negative culture
2	Severe soft tissue swelling; periosteal reaction of distal scapula and proximal	N/A	N/A	NA	Mononuclear Inflammation (large mononuclear cells predominate)
					Negative culture
					Lymphoplasmacytic synovitis with fibrosis
3	Sclerosis within the insertion of the teres, infraspinatus, and supraspinatus; large caudal glenoid fragment; bone spur off caudal humeral head; flattening of glenoid cavity	Fibrous scar tissue/adhesions	N/A	Collapsed joint capsule; severe capsular adhesion; grade IV/IV cartilage erosion of humeral head; MUA performed, abrasion arthroplasty performed, caudal glenoid fragment removed	N/A
4	Normal	Significant fibrous/periosteal reaction around the whole shoulder joint, including the biceps groove and infraspinatus insertion area	Marked shoulder effusion; severe inflammation of the shoulder joint capsule	N/A	Mononuclear inflammation (large mononuclear cells predominate)
					Negative culture
5	Remodeling and sclerosis within the bicipital groove; osteophyte off the caudal humeral head and glenoid cavity	Bilateral supraspinatus tendinopathy	N/A	Significant adhesions and fibrosis surrounding biceps tendon (debrided); severe supraspinatus bulge; severe synovitis; grade IV/IV cartilage lesions on humeral head. Abrasion arthroplasty performed, RF performed to all areas of inflammation and disruption	N/A
6	Sclerotic rim at the region of insertion of the capsule and tendons at the proximal humerus/humeral head	Severe disruption and insertionopathy of the right biceps muscle/tendon unit; right supraspinatus tendinopathy; moderate disruption and insertionopathy of the left biceps tendon	Right shoulder muscular atrophy, severe biceps tendinopathy, mild shoulder effusion	Significant inflammation of the glenohumeral ligament, synovium, subscapularis tendon and joint capsule; mild bulge of the supraspinatus creating impingement of the biceps	Chronic sterile histiocytic inflammation, low-to-moderate grade chronic hemorrhage
7	Severe remodeling, sclerosis, and collapse in joint space; periosteal reaction along the proximal humerus and distal scapula	N/A	Severe joint inflammation and marked enhancement of the joint capsule	Shoulder joint collapsed; unable to identify normal anatomy; grade IV/IV cartilage erosion caudal humeral head. Abrasion arthroplasty performed and MUA performed	N/A
8	Severe osteoarthritis; irregular new bone proliferation in the region of the bicipital groove; less severe changes in the contralateral shoulder	Chronic biceps tendinopathy and bursitis, bilateral teres minor chronic tendinopathy	Right shoulder dysplasia with severe osteoarthritis, synovitis and joint capsule thickening; biceps tenosynovitis; mild supraspinatus, infraspinatus, and teres minor insertionopathy. Mild left shoulder dysplasia	N/A	N/A

### Radiographs

**Figure 1:**
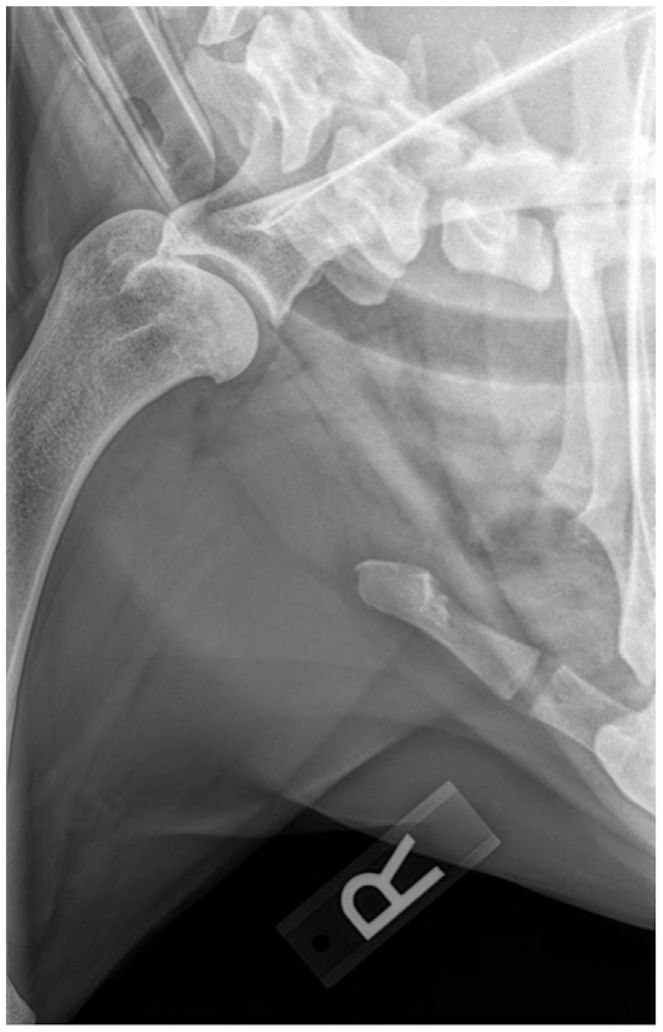
**A right lateral shoulder radiograph of a patient diagnosed with adhesive capsulitis**. There is a sclerotic rim at the region of insertion of the capsule and tendons at the proximal humerus and humeral head.

Routine lateral and caudocranial radiographs of the shoulders were performed in all eight dogs.^2^ Radiographs were unremarkable in two of the eight dogs. Radiographs of the affected shoulder revealed severe soft tissue swelling and periosteal reaction of the distal scapula and humerus in one dog. Radiographs of the affected shoulder revealed sclerosis within the insertion of the capsule, teres, infraspinatus, and supraspinatus in four patients. Two patients were noted to have remodeling and sclerosis within the bicipital groove. Radiographs of the affected shoulder revealed severe remodeling, sclerosis, and collapse in the joint space in two patients (Figure 1). There was a large caudal glenoid fragment noted in one patient. Three patients were noted to have osteophytosis of the caudal humeral head with flattening of the glenoid cavity. Radiographs of the unaffected shoulder and both elbows were performed in all patients and did not have any significant findings.

### Diagnostic Ultrasound

**Figure 2:**
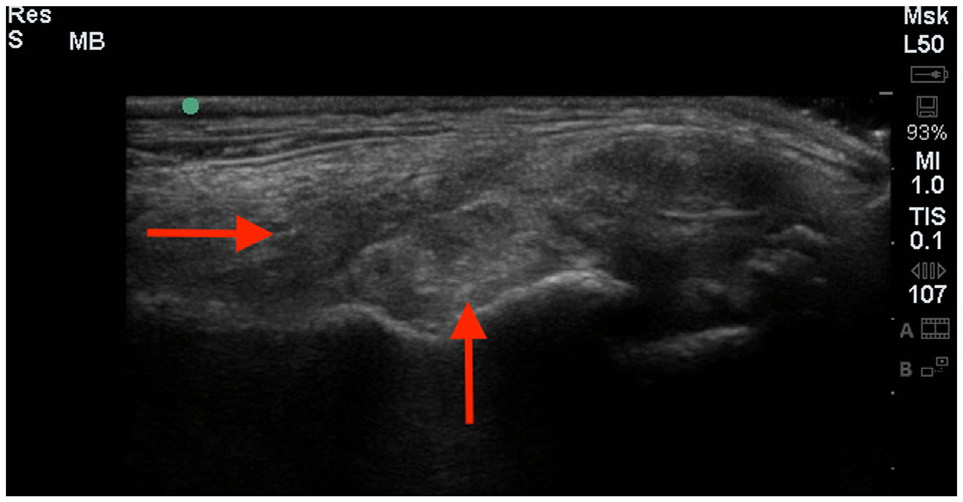
**A longitudinal diagnostic ultrasound of the shoulder revealed evidence of significant fibrous scar tissue and disruption of the supraspinatus fiber pattern (red arrows)**.

**Figure 3:**
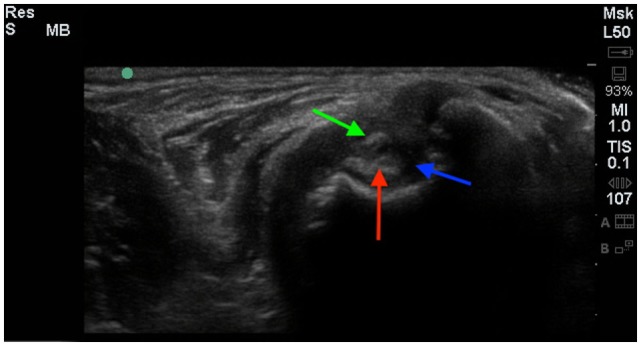
**A cross-sectional diagnostic ultrasound of the shoulder revealed evidence of significant fibrous scar tissue (green arrow), shoulder effusion (blue arrow), and hyperechoic changes of the biceps (red arrow)**.

**Figure 4:**
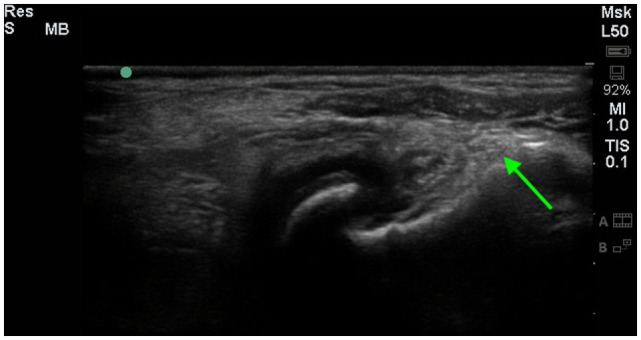
**A longitudinal diagnostic ultrasound image of the shoulder revealed evidence of significant fibrous/periosteal reaction around the whole shoulder joint, including the infraspinatus insertion area (green arrow)**.

Six of the eight dogs had an initial diagnostic ultrasound of the shoulder that revealed evidence of fibrous scar tissue in all six dogs (Figures 2–4).^3^ A diagnostic ultrasound of the affected shoulder in one patient revealed significant disruption of the supraspinatus fiber pattern, with shortened, irregular fibers at the insertion. In three of the six patients, diagnostic ultrasound of the affected shoulder showed an intact and overall normal supraspinatus insertion/origin. In the other three patients the supraspinatus was noted to have areas of hypoechoic and hyperechoic foci with a non-homogenous fiber pattern. In one patient, the supraspinatus appeared to impinge upon the biceps tendon. In one patient, there was a significant hyperechoic calcified foci impinging upon the biceps groove in addition to a hyperechoic periosteal reaction noted at the supraglenoid tubercle at the biceps origin. In three patients, significant fiber disruption was noted at the point of origin of the biceps, as well as large areas of hypoechogencity and periosteal reaction at the supraglenoid tubercle. There were changes in the surrounding connective tissue and infraspinatus and teres tendons, indicating fibrous scar tissue/adhesions in three patients. The joint capsule appeared hyperechoic, disrupted, and thickened in two patients. There was a moderate amount of shoulder joint effusion present in addition to free floating fibrous tissue noted in one patient.

### Magnetic Resonance Imaging

**Figure 5:**
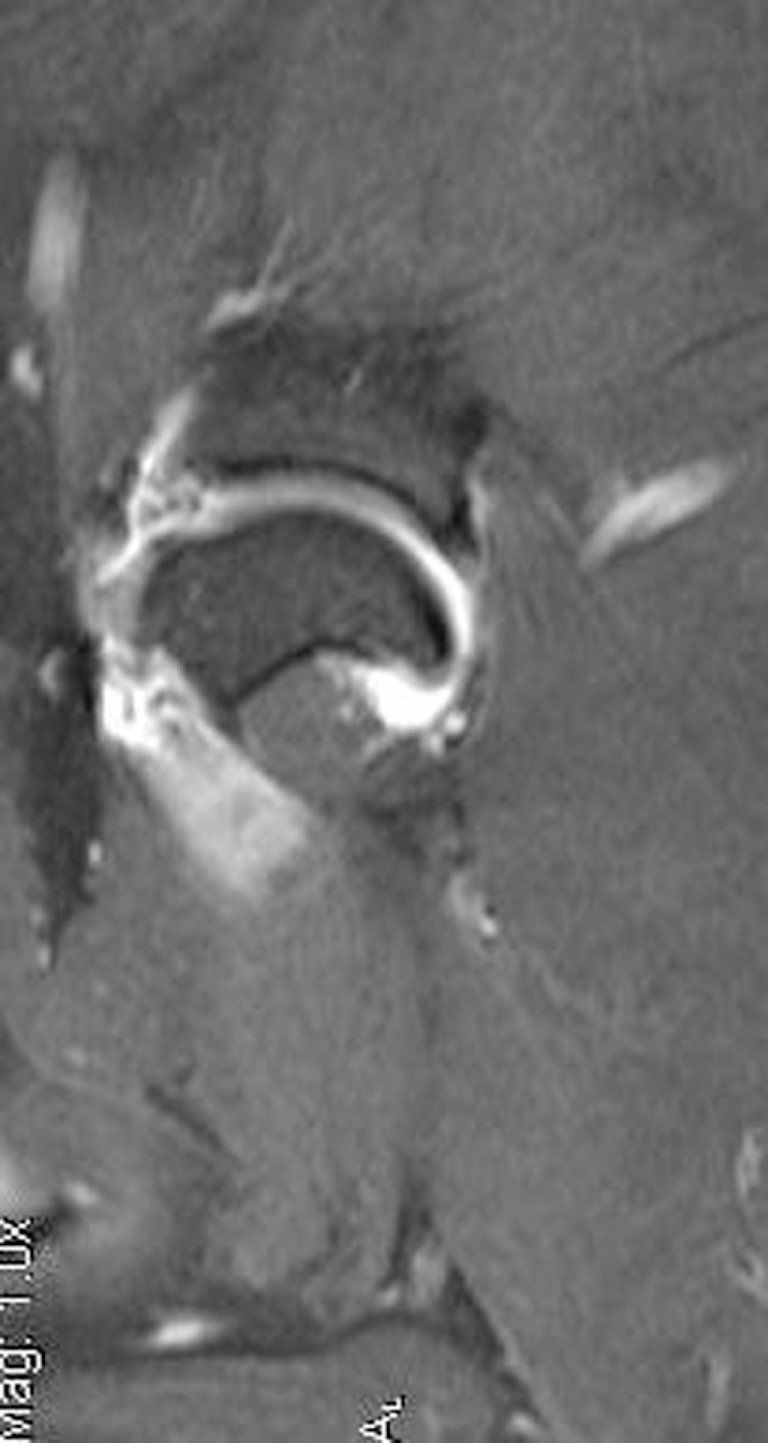
**This is an MRI T2 sagittal view of a patient diagnosed with adhesive capsulitis**. There is moderate shoulder effusion and enhancement of the synovial lining of the shoulder, including the portion of the joint capsule that envelops the biceps tendon was present.

Magnetic resonance imaging was recommended in all patients to evaluate the soft tissue structures of the shoulder, particularly the joint capsule and supraspinatus tendon, which are commonly affected in human patients with adhesive capsulitis. Five of the eight patients elected to pursue MRI. Marked muscle atrophy of the shoulder musculature was noted in all five patients that had MRI of their cervical spine and affected shoulder. Moderate shoulder effusion and enhancement of the synovial lining of the shoulder, including the portion of the joint capsule that envelops the biceps tendon was appreciated in four of the five patients (Figure 5). Findings revealed a severe biceps tendinopathy with mild shoulder effusion in the last patient. One patient was noted to have damage to the supraspinatus tendon and medial glenohumeral ligament and another patient had signs of severe shoulder dysplasia with severe osteoarthritis, synovitis, and joint capsule thickening in addition to mild supraspinatus, infraspinatus, and teres minor insertionopathy. Mild enlargement of the axillary lymph node was noted in two of the patients.

### Arthroscopy

**Figure 6:**
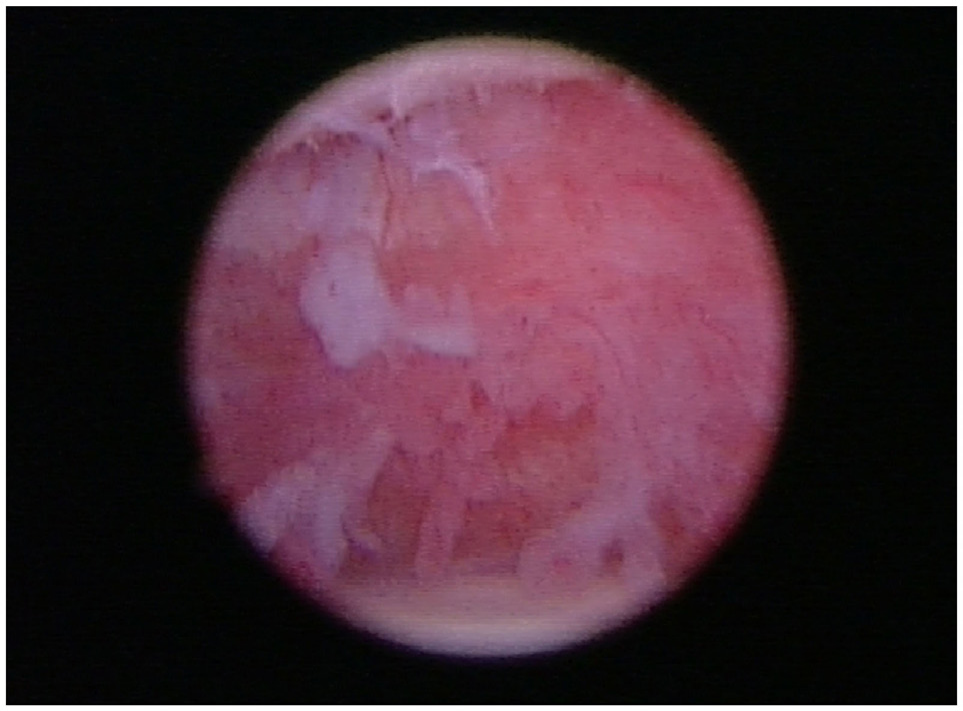
**In all patients, arthroscopy of the affected shoulder revealed the synovium was inflamed and disrupted**.

**Figure 7:**
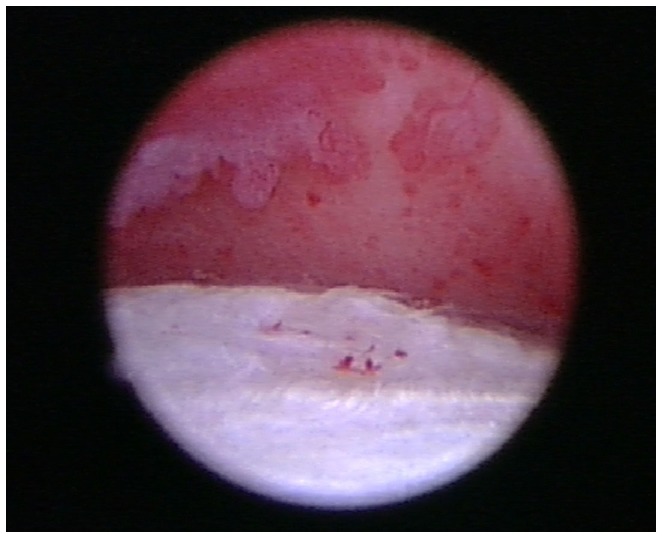
**Areas of grade III/IV cartilage erosion on Modified Outerbridge Cartilage Scoring System were noted along the caudal humeral head and glenoid during arthroscopy in this patient diagnosed with adhesive capsulitis**.

Shoulder arthroscopy was performed in five of the eight patients. A standard lateral portal was utilized for arthroscopic evaluation of the affected shoulder. Following aseptic preparation, a 22-gage needle was placed into the shoulder. Synovial fluid was aspirated using a 5 ml syringe to ensure the needle was in the joint. Approximately 5–10 ml of saline was then injected into the shoulder to distend the joint. A #15 blade was used to create a stab incision through the skin to allow for insertion of the cannula and trochar into the shoulder. The 1.9 mm arthroscope was then inserted into the cannula. The shoulder was then assessed. A significant amount of scar tissue and adhesions were diffusely noted in three patients. The synovium was inflamed and disrupted in all patients, but severe inflammation was noted throughout the synovium of two patients (Figure 6). Moderate-to-severe inflammation of the biceps tendon and a moderate bulge of the supraspinatus, creating significant impingement of the biceps tendon was noted in one patient. Mild-to-moderate fraying and disruption of the subscapularis, medial glenohumeral ligament, and medial compartment/joint capsule was noted. Areas of grade III–IV/IV cartilage erosion on Modified Outerbridge Cartilage Scoring were noted along the caudal humeral head and glenoid in three patients (Figure 7). In these patients, abrasion arthroplasty was then performed. A large caudal glenoid slab fracture was noted and subsequently removed in one patient. In one patient, the entire joint space had collapsed, and the scapular-humeral joint appeared to have ankylosed through soft tissue contracture. In this patient, no normal anatomy could be identified at the time of arthroscopy. All incisions were routinely closed.

One patient had a second-look arthroscopic exploration of the affected shoulder 8 weeks following ADPC–PRP therapy, which revealed severe inflammation and tenosynovitis of the biceps tendon, with inflammation evident within the bicipital groove. The tendon had an “hour glass” appearance, and when probed, an area of irregularity and weakness in the fiber pattern was appreciated. Arthroscopic release of the tendon was performed. The previously noted bulge of the supraspinatus was significantly improved. The medial glenohumeral ligament and subscapularis were significantly improved, and areas of neovascularization/new blood supply were noted. Fibrocartilaganous growth was noted on the caudal humeral head and glenoid cavity. Significant synovitis was still noted.

### Cytology and Histopathology

Arthrocentesis of the affected shoulder was performed in four of the eight dogs and submitted for cytology. Cytology results were consistent with mononuclear inflammation (large mononuclear cells predominated) in three dogs and chronic sterile histiocytic inflammation with low grade to moderate and low-grade chronic hemorrhage in one dog. Histopathology of the shoulder synovium was performed in one patient and consistent with marked lymphoplasmacytic synovitis with fibrosis.

### Conservative Management

All eight patients tried conservative management (Table 4). Seven patients were prescribed a non-steroidal anti-inflammatory medication (carprofen 2 mg/kg by mouth every 12 h, deracoxib 1–2 mg/kg by mouth every 24 h, or meloxicam 0.1 mg/kg by mouth every 24 h) and tramadol (2–4 mg/kg by mouth every 8 h). The other patient was prescribed codeine after a course of tramadol. Five dogs were prescribed gabapentin (5 mg/kg by mouth every 12 h). Four dogs were prescribed methocarbamol (15 mg/kg by mouth every 12 h). Three dogs were prescribed traumeel, a homeopathic, anti-inflammatory agent used to treat inflammation and pain (2 tablets by mouth every 8 h). All patients were directed to take a glucosamine/chondroitin sulfate joint supplement and omega-3 fatty acid supplement. Two patients were prescribed a course of prednisone (1 mg/kg by mouth every 24 h). Three patients received an intra-articular corticosteroid injection (methylprednisolone acetate, 20 mg). Seven patients participated in weekly rehabilitation, consisting of manual therapy (massage, stretching, passive range of motion, joint manipulation), therapeutic ultrasound/transcutaneous electrical nerve stimulation (TENS) combination therapy (frequency 2 MHz, intensity 0.5 pps, 5 min, duty 50%), laser/LED therapy to the carpi, elbows, supraspinatus, shoulders, scapula, and paraspinal musculature (dose: 5 J/cm^2^), and magnetic field therapy.^4^ Five of the eight dogs elected to try underwater treadmill hydrotherapy, but discontinued after two sessions due to worsening of lameness. Owners elected amputation in one patient after the patient did not respond to conservative management with medical therapy and rehabilitation therapy. Five of the eight dogs received three sessions of extracorporeal shockwave therapy (ESWT) at 2-week intervals (3.54 mJ applied at 0.14 mJ/mm^2^, 240 shocks/min, 1000 shocks).^5^ Slight improvement in lameness was noted in two of the five patients that received ESTW; however, the other three did not show significant improvement. Both patients that showed improvement originally presented with a grade V–VI/VI unilateral forelimb lameness, and after three sessions of ESWT at 2-week intervals, the patients had improved to a grade IV/VI unilateral forelimb lameness at a recheck examination 2 weeks after finishing ESWT.

**Table 4:** **Treatments performed**.

Patient	LLLT	Therapeutic ultrasound	ESWT	UWTM	IA steroids	Regenerative medicine	Amputation
1	Yes	Yes	Yes	Yes	Yes	IA ACS/ADSC	No
						IT PRP/ADSC	
2	Yes	Yes	Yes	No	Yes	No	Yes
3	Yes	Yes	Yes	Yes	No	No	No
4	Yes	Yes	Yes	No	No	No	No
5	Yes	Yes		Yes		IA ACS/ADSC	
						IT PRP/ADSC	
6	Yes	Yes	No	Yes	No	No	No
7	Yes	Yes		Yes	Yes	IA ACS/ADSC	
8	No	No	Yes	No	No	No	No

### Stem Cell Therapy

Three patients received an intra-articular injection of ADPC and ACS in the affected shoulder and two of them received an ultrasound-guided ADPC and leukocyte-poor PRP injection in the affected supraspinatus (see text footnote 1) (15–17). A diagnostic ultrasound re-evaluation was performed at 4 and 8 weeks following regenerative medicine therapy. At 4 weeks, the supraspinatus tendon appeared to have a more organized fiber pattern and no impinge upon the biceps tendon as previously noted in two patients. However, both patients still had moderate shoulder joint and bicipital sheath effusion. At 8 weeks following regenerative medicine therapy, neither patient’s lameness nor diagnostic ultrasound findings had improved. One patient elected to pursue a second ultrasound-guided ADPC–PRP injection in the affected supraspinatus and ADPC–ACS in the affected shoulder was performed. A recheck diagnostic ultrasound of the affected shoulder was performed 4 weeks later in this patient. Findings in the affected shoulder revealed chronic fibrous changes were still present. There also appeared to be an area of fibrous contracture developing within the infraspinatus and teres tendons, which were previously noted as normal. Eight weeks later, no improvement in lameness or diagnostic ultrasound findings was noted. A third ultrasound-guided ADPC–PRP injection was injected into the supraspinatus tendon bilaterally and ADPC–ACS were injected into the affected shoulder. A recheck diagnostic ultrasound of the shoulder was performed 8 weeks later, which revealed the supraspinatus fibers appeared more organized and homogeneous. However, a significant amount of hyperechoic chronic fibrous and periosteal changes noted along the biceps groove. The joint capsule appeared unorganized, hyperechoic, and thickened. The teres tendon and infraspinatus insertion point appeared remodeled and showed hyperechoic fibrous tissue similar to prior scans. The area identified as a firm area within the muscle belly of the infraspinatus on palpation and restricting full shoulder extension on manual palpation remained unchanged. Overall, no improvement was appreciated on diagnostic ultrasound in spite of regenerative medicine therapy in this patient.

### Follow-Up

Three of the eight dogs were lost to follow up. A mean of 20.5 months follow-up was available (range 12–29 months). Five owners were asked to fill out a questionnaire and Canine Brief Pain Inventory at the time of re-evaluation. None of the five dogs were reported to have any improvement since their last visit at VOSM. One of the four dogs had died of an unrelated cause. Four of the five dogs were still said to be non-weight bearing the majority of the time in the affected limb. One dog was reported to have a mild weight bearing lameness, which worsens after 15 min of walking activity. None of the owners felt that any of the therapies or treatments attempted helped; however, two owners reported that their dog appeared to be subjectively more comfortable immediately following rehabilitation therapy. Three of the five owners felt that having a frozen shoulder significantly interfered with their dogs gait, general activities, and enjoyment of life.

## Discussion

Despite anatomical difference between human and canine shoulders, orthopedic examination findings in the eight patients appear to be similar to those of human patients with adhesive capsulitis. Human patients present with restricted ROM, pain on shoulder manipulation, and impaired function. Similarly, all eight of the patients initially presented with a chronic, grade III–VI/VI unilateral forelimb lameness and had moderate to significant discomfort on and restriction of shoulder extension and flexion. While intra-articular anesthesia is commonly used in humans at the time of diagnosis, this was not performed at the time of initial consult in the patients included in this study due to the need for sedation. However, all patients were sedated and/or anesthetized for either diagnostics or treatment, at which time shoulder extension and flexion were confirmed to be restricted.

Cytologic and histopathologic findings were consistent with findings appreciated in human patients with adhesive capsulitis in the four patients who pursued these diagnostics. Three of the eight patients had a history of previous trauma to the affected limb. This could suggest that this patient had adhesive capsulitis secondary to chronic inflammation and instability. However, while the other five patients had no history of trauma, all patients had a history of a chronic, unilateral forelimb lameness. It is unclear in these cases whether the adhesive diagnosis is primary or potentially secondary to a chronic, untreated shoulder pathology. In the authors’ opinion, just as in humans, adhesive capsulitis may be the end-stage manifestation of several primary conditions, it seems possible that regardless of the etiology of the primary shoulder pathology, chronic, untreated shoulder pathology could lead to adhesive capsulitis (2). Further study is necessary to confirm this. Additionally, three of the eight patients in this study were Corgis. Future studies should be performed to assess if there is either a breed predilection or congenital condition that predisposes patients to developing adhesive capsulitis.

While adhesive capsulitis in humans is usually a clinical diagnosis made upon review of patient history and assessment of range of motion before and after injection of local anesthetic, diagnostic imaging is performed to rule out other differential diagnosis and fully evaluate the shoulder. Radiographs of the shoulder are performed to rule out other comorbidities, including fracture, dislocation, calcific tendonitis/bursitis, acromioclavicular arthrosis, and abnormal acromial morphology. CT would have been required to further evaluate the cartilage and boney structures of the joint. However, in this case series, MRI was recommended over CT to evaluate the soft tissue structures of the shoulder, which was the primary area of interest. MRI is routinely performed in humans with suspected frozen shoulder to evaluate the soft tissue structures of the shoulder, including the joint capsule, rotator cuff, biceps, glenohumeral, and coracohumeral ligaments. Shoulder effusion and enhancement of the synovial lining of the shoulder, including the portion of the joint capsule that envelops the biceps tendon, has been documented in human patients with frozen shoulder (18). One recent study also reported that 62% of human patients with idiopathic adhesive capsulitis were found to have concurrent supraspinatus lesions on MRI (19). Four of the five patients who had an MRI were found to have moderate shoulder effusion and enhancement of the synovial lining of the shoulder, which has also been documented in human patients with frozen shoulder (18). One patient was also found to have MRI findings consistent with supraspinatus tendinopathy in the affected limb and another was found to have signs of supraspinatus, infraspinatus, and teres minor insertionopathy, which have also been reported in humans.

Diagnostic ultrasound is also used to evaluate the soft tissue structures of the shoulder in patients with suspected frozen shoulder. Findings that have been seen in patients with adhesive capsulitis are enhanced vascularity and hypoechoic change around the rotator interval (20, 21). Dynamic sonography has been used to help diagnose adhesive capsulitis, which reveals a limited but continuous sliding movement of the supraspinatus tendon against the acromion of the scapula (22). Six of the eight dogs had an initial diagnostic ultrasound performed, all of which revealed evidence of effusion, thickening of the synovium, and fibrous scar tissue. Three dogs were noted to have changes consistent with supraspinatus and biceps tendinopathy. Two dogs had recheck diagnostic ultrasounds performed after receiving ultrasound-guided ADPC and PRP injection in the supraspinatus and ADPC and ACS in the affected shoulder. While the fiber pattern of the supraspinatus was noted to be more regular and homogeneous, moderate inflammation and fibrous changes were still appreciated.

Arthroscopy was performed in five of the eight patients. Just as in human patients with adhesive capsulitis (23), significant adhesions, fibrous scar tissue of the joint capsule, severe inflammation throughout the synovium, severe soft tissue contracture, and loss of capsular volume were found, all of which are very characteristic findings in human patients with adhesive capsulitis.

Treatment offered to human patients with adhesive capsulitis depends on the stage of the disease and clinical symptoms. Frozen shoulder was originally described as a self-limiting disease, and supervised neglect with analgesia, supervised physical therapy, and/or intra-articular corticosteroid injections were recommended (24). Recent studies report that 90% of cases resolve with conservative therapy, which often includes non-steroidal anti-inflammatory medications, intra articular corticosteroid injections, oral steroids, and physical therapy and rehabilitation (6). Intra-articular corticosteroid injections are commonly used and have been reported to have similar outcomes to physiotherapy alone and to more invasive measurements, such as manipulation and hydrodilation (25–27). Moreover, a double-blind, sham-controlled randomized study demonstrated that corticosteroid injections were better than a sham injection in improving shoulder pain at 6 weeks, and the effect was maintained for 12 weeks (28). Interestingly, one recent prospective study compared intensive physical therapy, including passive stretching and manual mobilization versus supportive therapy and exercises with pain limits, or “supervised neglect” and found that patients receiving intensive physical therapy did not do as well as those treated with supervised neglect (29).

In this case series, conservative management consisting of oral medications, intra-articular depomedrol injection, laser/LED therapy, massage therapy, therapeutic ultrasound, magnetic field therapy, and hydrotherapy did not appear to have a significant clinical effect. Of the treatment modalities performed, ESWT seemed to be the most effective treatment modality. Of the five that received ESWT, two showed clinical improvement in lameness. Those dogs originally presented with a grade V–VI/VI unilateral forelimb lameness, and after three sessions of ESWT at 2-week intervals, the patients had improved to a grade IV/VI unilateral forelimb lameness. Two patients received ultrasound-guided ADPC and PRP injection in the affected supraspinatus, and ADPC and ACS in the affected shoulder. While the supraspinatus fibers appeared more homogeneous and organized on 4-week recheck diagnostic ultrasound, suggesting that the supraspinatus tendinopathy had improved, both cases still had evidence of severe, chronic fibrous changes present within the joint and joint capsule. There are currently no reports of the use of stem cell or PRP therapy for adhesive capsulitis in humans.

While conservative management is successful in many of human cases, if there is little to no improvement, surgery may be indicated (7). A recent prospective, randomized study, which compared arthroscopic capsular release to a gentle home stretching program, demonstrated both treatment options to be effective treatment modalities (30). Goals of surgery are to decrease pain, improve ROM, improve function, and shorten natural history of disorder (1, 31–33). The mainstay of surgical treatment is to perform arthroscopic capsular release (1, 2, 31–33). Postoperative care regiments generally include starting physical therapy within 1–2 days of surgery, and patients are encouraged to participate in a physical therapy program for 8–10 weeks (1). Recent studies suggest more patients have pain free range of motion around 10 weeks postoperatively (33). Moreover, patients reported excellent outcome following an arthroscopic anterior capsule and rotator interval release (34). To the authors’ knowledge, there are currently no reports documenting surgical treatment of this condition in dogs. Further study is needed to assess the efficacy of surgical management of adhesive capsulitis in dogs.

Adhesive capsulitis is an uncommon cause of chronic forelimb lameness. Due to the rare nature of this condition, this case series only describe eight patients. While the initial presentation of adhesive capsulitis in dogs is very similar to that in humans, the progression, response to therapy, and outcomes found in this study were not (35, 36). Recent studies performed in humans show that adhesive capsulitis often resolves with conservative management (6, 25–29, 36). However, none of the patients treated had complete resolution or significant improvement, regardless of the therapy elected. It is also important to note that while many of these patients had evidence on diagnostic imaging (diagnostic ultrasound or MRI) of concurrent supraspinatus, biceps, and/or infraspinatus tendinopathies, the progression, response to therapy, and outcomes are also not consistent with a primary supraspinatus, biceps, and/or infraspinatus tendinopathy. Further investigation is needed to further describe the etiology and pathogenesis of adhesive capsulitis in dogs. Future controlled, randomized, and blinded investigations with validated objective outcome measures are also warranted to evaluate the effectiveness of both non-surgical and surgical treatment modalities, establish treatment protocols, and evaluate short- and long-term clinical outcome of patients.

## Ethics Statement

In accordance with AAALAC International Rules of Accreditation, this retrospective study was performed with the approval of the VOSM Research Committee and with owner consent. In this retrospective study, all dogs whose records were reviewed were client owned dogs. All clients volunteered their dog for the study and provided written consent as required by Veterinary Orthopedic and Sports Medicine Group for every study participant.

## Author Contributions

All authors have made substantial contributions to the conception or design of the work. All authors have participated in the acquisition, analysis, or interpretation of data for the work. All authors have contributed to the drafting the work and revising it. All authors have consented final approval of the version to be published and agreed to be accountable for all aspects of the work in ensuring that questions related to the accuracy or integrity of any part of the work are appropriately investigated and resolved.

## Conflict of Interest Statement

The authors certify that they have no affiliations with or involvement in any organization or entity with any financial interest, or non-financial interest in the subject matter or materials discussed in this manuscript.

## Footnotes

^1^Regenerative Medicine Service, Marion DuPont Scott Equine Medical Center, Virginia-Maryland Regional College of Veterinary Medicine, Leesburg, VA, USA.

^2^SoundEklin, Sound™, Carlsbad, CA, USA.

^3^Hitachi-Aloka, Aloka, Wallingford, CO, USA.

^4^The Dynatron Solaris 708, Dynatronics, Salt Lake City, UT, USA.

^5^VersaTron 4 Paws Trode, PulseVet, Alpharetta, GA, USA.

## Abbreviations

ADPC, adipose-derived progenitor cell; PRP, platelet-rich plasma; ACS, autologous conditioned serum; ESTW, extracorporeal shockwave therapy.
